# Understanding Immune Responses to Lassa Virus Infection and to Its Candidate Vaccines

**DOI:** 10.3390/vaccines10101668

**Published:** 2022-10-06

**Authors:** Hannah Murphy, Hinh Ly

**Affiliations:** Comparative & Molecular Biosciences Graduate Program, Department of Veterinary & Biomedical Sciences, College of Veterinary Medicine, University of Minnesota, Twin Cities, St. Paul, MN 55108, USA; murp1625@umn.edu

**Keywords:** Lassa virus, arenavirus, mammarenavirus, pathogenicity, pathogenesis, virulence, vaccine, therapeutics, diagnostics, hemorrhagic fevers

## Abstract

Lassa fever (LF) is a deadly viral hemorrhagic fever disease that is endemic in several countries in West Africa. It is caused by Lassa virus (LASV), which has been estimated to be responsible for approximately 300,000 infections and 5000 deaths annually. LASV is a highly pathogenic human pathogen without effective therapeutics or FDA-approved vaccines. Here, we aim to provide a literature review of the current understanding of the basic mechanism of immune responses to LASV infection in animal models and patients, as well as to several of its candidate vaccines.

## 1. Introduction

Lassa virus (LASV) causes an acute infectious disease in humans known as Lassa fever (LF), a hemorrhagic fever disease, which is endemic to West Africa. Although endemic to several West African countries, a significant number of travel-associated LF cases have been recorded worldwide, including in the United States, Europe, and Asia. These travel-associated cases of LF have been summarized in a recent review article [1]. While it is probably undercounted due to the lack of standardized surveillance for LASV, it has been estimated that 100,000–300,000 LASV infections occur each year and can result in about 5000 deaths annually [2]. Because there are no standardized surveillance mechanisms in place, the number of LASV cases in West Africa is likely underreported. Among all known viral hemorrhagic fevers, LF has the largest global burdens, second only to Dengue fever (which has approximately 390 million illnesses per year) [3,4,5]. Most LASV-infected people can successfully establish an immune response that is strong enough to control the infection, but some people develop severe LF that ultimately results in death.

There are currently no FDA-approved vaccines or effective therapeutics against LASV [6]. The high genetic variability between LASV strains and the reported virus-mediated immune evasion mechanisms hamper the development of effective prophylactic and therapeutic modalities. As a result, the World Health Organization (WHO) has placed LF on their Blueprint list of priority diseases which require a greater level of understanding. A better understanding of LASV virulence and LF disease pathogenicity is needed to develop proper preventative, therapeutic, and diagnostic methods. Toward this end, this review aims to provide a literature review of the current understanding of the basic mechanism of immune responses to LASV infection in animal models and human patients, as well as to several candidate vaccines that have been developed for LF. 

## 2. LASV Transmission and LF Disease Pathogenesis

There are multiple ways that LASV can spread to people, with rodent-to-human transmission (resulting from close contact with infected rodents or their excrement) being the most prevalent means of virus transmission [7]. Although the most common mode of LASV transmission is rodent-to-human, 20% of LF cases are thought to be due to human-to-human transmission [8]. In its natural animal reservoirs, such as multimammate rodent species, such as *Mastomys natalensis*, *Mastomys erythroleucus*, *Hylomyscus pamfi*, *Praomys daltoni*, *Mus baoulei*, *Rattus rattus*, *Crocidura* spp., *Mus minutoides*, *and Praomys misonnei* [9,10,11], LASV is thought to cause an asymptomatic infection. However, like in the case of humans, certain strain(s) of LASV can cause symptomatic disease in experimentally infected rodents including *Mus musculus* (house mouse), *Rattus rattus*, *Rattus fuscipus*, and the *Myosoricinae soricidae* (shrew) [9,10].

The prevalence of LF is unknown since currently available estimates are based on studies in the 1980s which used relatively primitive clinical diagnostics and surveillance methods by today’s standards. The significant variability of LF symptoms and lack of proper standardized diagnostics lead scientists to think that the actual annual LF case numbers are much higher than the current estimate of 100,000 cases per year [12]. The Coalition for Epidemic Preparedness Innovations (CEPI) is investigating the incidence of the LF disease in Nigeria and several of its surrounding endemic nations in West Africa to acquire a better understanding of the true frequency of the disease. Based on their data and the epidemiology of LF, the United National Development Programme calculates that 59 million seronegative people are at risk of LASV infection. This was based on information regarding annual infections, annual seroconversions of LASV, and annual ratios of mortality from LASV infection [13].

Following exposure, LF has an incubation period of approximately 7–21 days. While the majority of patients are asymptomatic, ~20% of LASV infections are symptomatic [14]. Early LF symptoms are often moderate and are also comparable to those of other febrile disorders which makes LF diagnosis challenging. Patients with fevers over 38 °C (100.4 °F), who either reside in LF endemic areas or have recently been traveling to recognized endemic areas, and who are not responding to antimalarial or antibiotic therapies, should be suspected of having LF [13]. Individuals with mild LF disease have reported influenza-like symptoms (e.g., fever, weakness, malaise, and headaches); however, individuals with severe LF disease experience acute and multisystem disease symptoms [14].

Early phase disease progression can commonly include joint and lower back pain, a nonproductive cough, and a sore throat [15]. Pharyngitis (yellow exudate patches that form on tonsils in a pseudomembrane), as well as diarrhea, vomiting, and abdominal discomfort affect about 70% of symptomatic patients [15,16]. In severe LF instances, increased vascular permeability is typical and can cause facial edema, pleural effusions, and pericardial effusions. [15]. Severe LF patients frequently have acute respiratory discomfort along with laryngeal edema and fluid buildup in lung cavities [15]. Mucosal bleeding with low blood pressure is present in 15%–20% of severe LF cases [15]. Recovery from mild LF disease occurs between 8–10 days after symptom onset; however, in severe LF disease cases, deterioration of patients is seen 6–10 days after symptom onset [15]. LASV viremia levels peak 4–9 days after symptom onset, and in patients who recover, the virus is cleared from the blood about three weeks after symptom onset [15]. A person who exhibits severe LF symptoms and a high level of viral load likely has an ineffective immune response to keep the viral dissemination under control and usually has a poor disease prognosis which can result in death [15]. Death usually occurs 14 days after reported symptom onset, following hypovolemic shock and signs of neurological disease (e.g., encephalopathy, disorientation, convulsions, comas, and seizures) [15].

LF is severe in pregnant women and is most severe in late pregnancy [17,18]. During early pregnancy, 92% of fetuses are spontaneously aborted from infected mothers, and 72% are aborted in the third trimester [18,19,20]. High viral loads in the placenta, fetal tissues, and maternal blood are associated with high risks of maternal and fetal mortality [17,18]. According to reports, pregnant women have a three times higher risk of dying from LASV infection than their non-pregnant peers [21]. Although there are LASV case reports with good maternal outcomes, it is rare for fetuses to survive the infection [17,22]. Diagnosing LF in children is more challenging (as compared to adults) since the disease manifestations are mild and nonspecific, which has led to LF not being often studied in children [15,23]. LF symptoms differ between neonates and older children. Neonates are commonly misdiagnosed with neonatal sepsis due to overlapping symptoms with LF (e.g., high-grade fever, swollen lymph nodes, convulsions, and hemorrhaging). LF symptoms in older children are more similar to adult LF symptoms (e.g., diarrheal disease, pneumonia, and prolonged fever) [15,23,24,25]. Poor indicators, such as hemorrhaging, acute renal failure, convulsions, and comas, are seen in children similar to adults with LF [26].

The majority of LF survivors experience neurological sequelae, which can include memory loss, ataxia, neuromuscular discomfort, and sensorineural hearing loss (SNHL). SNHL (bilateral or unilateral) is found in about 34% of LF survivors, and hearing loss is irreversible in 67% of cases [27,28,29]. Due to the potential underestimation of its incidence, SNHL is viewed as a neglected public health and socioeconomic burden in LF-endemic nation [27]. SNHL is characterized by cochlear hair cell damage and a hearing loss of 30 dB or higher over three different auditory frequencies [30]. SNHL typically appears late in the acute phase or early in the convalescence phase of the course of LF illness [31]. The mechanisms of hearing loss in LF survivors are not completely understood despite a definitive correlation between SNHL and LF infection. 

## 3. LASV Genomic Structure and Mechanisms of Immune Responses to and Evasion against LASV Infection

**Figure 1:**
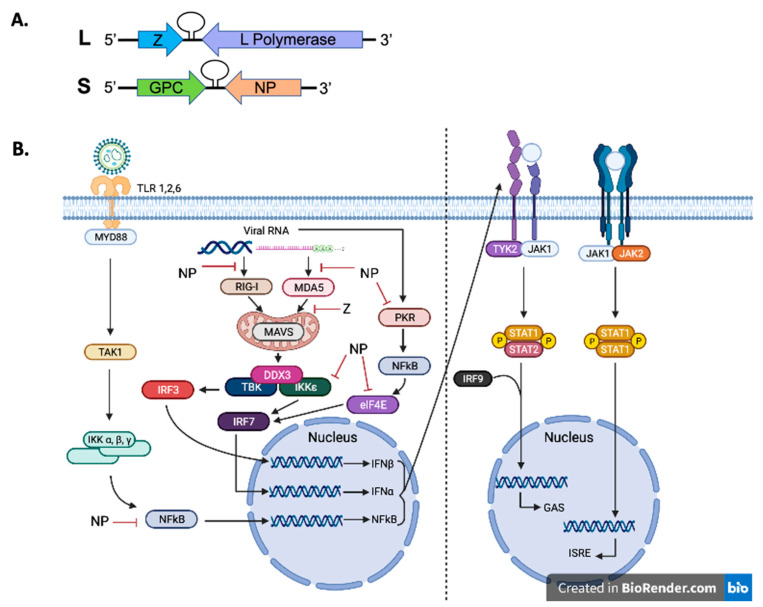
Arenaviral RNA genome structure and inhibition of innate immunity. (**A**) The genome of arenaviruses (e.g., LASV) is bisegmented and contained two genomic RNA segments that encode for four viral proteins in an ambisense coding strategy. Each of the segments have two open reading frames that encode for two gene proteins and is separated by a noncoding intergenic region (IGR). The IGR forms a stable hairpin RNA structure(s). The L segment (~7.2 kb) encodes for the L protein and Z protein. The S segment (~3.5 kb) encodes for the viral glycoprotein complex (GPC) and NP proteins. (**B**) The cellular Toll-like receptors (TLRs) recognize pathogen-associated molecular patterns (PAMPS), for example viral glycoproteins, and utilize the MYD88 adaptor protein to transmit cellular signals to influence the activities of other cellular proteins (e.g., TAK1, IKKa, β, and γ in order to activate transcription factors (e.g., interferon regulatory factors–IRF3 and 7) and NFkB subunits to induce the expression of IFNa and IFNb. To further activate other protein kinases (like TYK2, JAK1, and JAK2) and transcription factors (like STAT1 and STAT2 by phosphorylation), IFNa and IFNb are secreted from the cells and bind to their receptors on the membrane surface of cells. This causes the translocation of transcription factors (e.g., STAT1 and STAT2) from the cytoplasm into the nucleus, where they activate the transcription of hundreds of antiviral genes through their promoters (e.g., GAS or ISRE). On the other hand, aberrantly generated viral double-stranded RNAs (dsRNAs) are recognized as PAMPs by intracellular receptors, such as RIG-1 and MDA5. These activated intracellular receptors then activate cellular proteins such as MAVS (mitochondria), DDX3, TBK, and IKKe, which in turn activate IRF3, 7, and NFkB transcription factors to upregulate IFNa and IFNb. Aberrantly generated dsRNAs can also activate PKR (another intracellular receptor), which in turn activates NFkB and affects the function of other cellular proteins like elF4E to increase the expression of IFNa and IFN.

The LASV has a single-stranded negative-sense RNA genome. The large (L) segment and the small (S) segment make up the bisegmented genome. The genome uses an ambisense coding scheme to encode four viral proteins [32] (Figure 1A). 

The two segments each have two gene products within the two open reading frames and is separated by a noncoding intergenic region (IGR) which forms stable hairpin RNA structure(s) that are thought to help terminate mRNA transcription [32]. The L segment (i.e., large segment) encodes for the L and Z proteins. The L protein is the RNA-dependent RNA polymerase which facilitates viral RNA transcription and replication. The matrix protein containing the zinc-finger motif, known as the Z protein, serves a variety of purposes during the viral life cycle. The Z protein helps regulate RNA synthesis and orchestrates viral assembly and budding. Additionally, the Z protein antagonizes the host type 1 interferon (IFN-1) system [33,34,35,36,37,38], which will be discussed in more detail below. 

The viral glycoprotein complex (GPC) and NP proteins are encoded by the S segment, also referred to as the small segment. The glycoprotein (GP) of LASV mediates cellular entry. The GP1 C terminal domain interacts with α-dystroglycan (the cellular entry receptor) and allows the viral particle to be internalized by the host cell and delivered to the late endosomes [39,40]. Inside the late endosomes, acidification causes a pH change and this releases the ribonucleoprotein (RNP) complex into the cellular cytoplasm, where viral RNA synthesis occurs [41]. The NP is the main structural component of the ribonucleoprotein (RNP), as it encapsulates the bisegmented viral genome. NP functions, together with the L protein, to support viral genomic RNA synthesis. NP is also an antagonist of the host innate immune response, which will be described in more detail below. Modulation of the host immune response is carried out by degradation of virus-associated double-stranded RNAs (dsRNAs), which act as pathogen-associated molecular patterns (PAMPs), via the exoribonuclease function of NP [42,43].

Lassa fever virus causes generalized immune suppression of both the innate and adaptive immune responses. During early infection, both macrophages and dendritic cells (DCs) are targeted; however, macrophages are the main cellular target of LASV [44,45,46,47]. After becoming infected, macrophages help LASV disseminate when they enter draining lymph nodes and subsequently return to the rest of the body [44,45,46,47]. LASV infection does not activate macrophages and DCs, and so, there is a lack of activation markers and cytokine expression (e.g., CD80, CD86, CD40, TNFα, IL1β, IL6, and IL12) [44,47,48]. The lack of macrophage and DC maturation causes a failure of proinflammatory cytokine secretion, and this leads to a failure of costimulatory molecule stimulation that is required for T cell proliferation and memory recall of adaptive immune cells to LASV and to its immunogenic antigens [45]. For example, in vitro experiments show that when an immature DC presents LASV antigens to T cells, it can induce a T cell tolerance condition rather than activation [48,49]. This tolerance can blunt T cell responses and inhibits the adaptive immune response. On the contrary, in LASV infection, an effective CD4 and CD8 T cell-mediated response is critical for recovery, but not B cell-mediated response [48,50]. It has been observed that patients who recover from acute LASV infection do not have a measurable neutralizing antibody response, and antibodies only develop in low titers late during the convalescence phase [51,52,53].

It is important to note that some LF survivors can develop polyfunctional CD8 and CD4 T cell responses that exhibit inter-lineage cross-reactivity [54]. This adaptive immunity is important in geographical areas where LASV lineages overlap [54]. To obtain important insight into the LASV-specific T-cell mediated immunity, Sullivan and colleagues generated a library of recombinant single-cycle replicating vesicular stomatitis viruses (VSV) which encoded the full-length and/or partial portions of the GPC and NP (Josiah strain, lineage IV) [54]. Using samples from 48 LF survivors (Nigerians and Sierra Leoneans), the authors quantified LASV-specific memory CD4 and CD8 T cell responses as determined by IFN-γ and TNF-α levels in samples compared to the unstimulated PBMCs [54]. It was shown that the antigens of lineage IV (Josiah strain) were being recognized by LASV-specific T cells that were generated during primary infections with lineage II and III LASV [54]. These results suggest that CD8 T cell responses can respond to different viral lineages to a relatively high degree and that the epitopes are likely located within some antigenic “hotspots” in the carboxy terminal regions of NP, GP1, and GP2. This cross-reactivity of T-cell mediated immunity to different lineages of LASV thus implies a significant functional level of adaptive cellular immunity in spite of the immunosuppressive effect during the early phase of the virus infection and suggests that high diversity among the different viral lineages does not necessarily abolish the putative conserved immune dominant T cell epitopes. It is noteworthy that we have recently shown that mice that lack the expression of one or both major cytosolic innate immune receptors (RIG-I and/or MDA5 knock out) can also exhibit a late but heightened level of adaptive (T-cell) immune responses to clear the infection by a prototypic arenavirus, called Pichinde virus (PICV), despite a defective level of innate immunity [55].

Vaccines that induce robust T cell responses against LASV glycoproteins are protective in non-human primates (NHPs) and guinea pigs against LASV challenge despite not inducing antibody responses, which suggest that antibodies might be less critical to confer protection [56]. LASV infection induces IgM and IgG production, but IgM/IgG titers do not correlate with clinical outcomes [57,58]. If LF patients (or experimentally infected animals) produce high levels of circulating activated T cells and chemokines (e.g., IL-8 and CXCL-10), they might possibly be able to recover from LASV infection. Conversely, low chemokines, T-cell levels, and delayed immune cellular activation often cause an individual (human or laboratory animal) to succumb to LF disease [52]. T cells seem to play a dual role during LASV infection, one that helps clear the infection, but when clearance of the virus is unsuccessful, they can facilitate disease and serve as a potential pathogenicity factor. Intuitively, it would be thought that T cells would be entirely beneficial since there is data correlating delayed T cell responses with poor outcomes in patients, but this does not appear to be the case. It has been shown that bystander T cells, that are generated after LASV infection, travel to inflamed tissues to cause tissue damage [59,60,61]. There is also evidence of fatal disease onset occurring when T cells (antigenically unrelated to LASV) are produced and when there is an early timepoint hyperexpansion of T-cell clones. It is currently hypothesized that nonspecific T-cell clones dominate the immune response and this can cause the patient to be unable to control viral replication, thus causing fatal disease onset [61,62].

T-cell defective mouse models and humanized xMHC-1 mice have both been used to study the dual role of T cells during LASV infection (i.e., clearing infection and potential pathogenicity factor). Unlike wild-type mice, the humanized animals are susceptible to LASV infection and cannot inhibit viral replication [59]. When depleted of T cells, the humanized MHC-1 mice had high viremia but did not develop disease. The lack of disease development suggests that T cells are essential for rapid viral clearance rather than disease pathogenesis. The lack of macrophage and DC activation (in mice that are depleted of T cells) suggests that T cell responses are responsible in part for deleterious inflammatory reactions as well as disease pathogenesis [59]. 

Although there is evidence suggesting that antibodies do not play as critical of a role in the control of LASV infection and recovery from the disease as T cells do, convalescent plasma and monoclonal antibody (mAb) treatments (derived from LASV survivors) have been shown to be protective in animal models (guinea pigs and NHPs) of LF as well as in some human LASV survivors [63,64,65]. For example, when convalescent serum of LASV-infected Hartley outbred guinea pigs was infused into the strain 13 inbred guinea pigs, 80%–100% of strain 13 guinea pigs could survive the viral challenge with LASV as compared to 0% when the untreated control serum was used [63]. The range of survival was dependent on varying doses and application times of the convalescent serum after infection with LASV. Additionally, Jahrling and colleagues have shown that sera from LASV-infected rhesus macaques and human LASV survivors could provide 100% and 80%–100% protection in rhesus macaques against LASV infection, respectively [64].

It is interesting to note that one of the first documented LF patients (nurse Lily “Penny” Pinneo) survived the infection after she received supportive care after being transferred from a hospital in Jos, Nigeria to a hospital in New York City [66]. Afterwards, two researchers at Yale University who were working to isolate LASV became infected. One researcher died, but the other (Dr. Jordi Casals) survived the infection after receiving blood donated from nurse Penny [66]. It is thought that the convalescent antibodies in the donated blood played an important role in his recovery, but it is also possible that the supportive care that Dr. Casals received also played a beneficial role. Additionally, it is possible that the infective agent had already become attenuated after serial infections. 

In addition to convalescent serum, monoclonal antibodies (mAbs), derived from long-term LF survivors have also been shown to be protective against LASV infection in guinea pigs and NHPs [65,67]. Robinson and colleagues identified 13 human mAbs (humAbs) from B cells of LF survivors which targeted the LASV glycoprotein and had neutralizing activity in in vitro studies [65,67]. Five of these humAbs showed 100% protection, two showed 90% protection, one showed 80% protection, and the other six showed only 20%–40% protection in Hartley outbred guinea pigs that were intraperitoneally challenged with LASV when they were treated with 30 mg/kg of the humAbs on day 0, 3, and 6 post challenge [65,67]. Subsequent testing with the most successful humAbs (i.e., 37.2D, 12.1F, 8.9 F, and 37.7 H) in cynomolgus macaques showed 100% survival when injected intravenously with 15 mg/kg at 0, 4, and 8 days post challenge [65,67]. Additionally, these authors also determined that the dominant class of neutralizing humAbs targeted the epitopes, which spanned both GP1 and GP2 subunits and that the binding of the nAbs required the critical interactions of the subunits in the prefusion state of the glycoprotein [67,68]. To the best of our knowledge, there are no human clinical trials using mAbs to treat LASV infection, but given the promising preclinical results in macaques, clinical trials for efficacy assessment in humans might be warranted. Additionally, the incorporation of both GP subunits in the prefusion state of the glycoprotein should be considered for incorporation into LF candidate vaccines to increase the likelihood of producing a protective antibody level.

The suppression of the type 1 interferon (IFN-1) system, which is predominantly comprised of IFNα and IFNβ expressions, is another method that LASV can exploit during infection to inhibit the innate immune system in addition to T cells (Figure 1B). The downstream adaptive immune responses must be coordinated through the IFN-1 system. To stop and fight the infection, the IFN-1 system must be activated. The observation of reduced IFN-1 expression in patients with severe LF coincides with the finding that mice lacking the IFN-1 pathway die from LASV infection following a virus challenge [53,69]. This mechanism is similar to what is seen in experimentally infected NHPs. Although IFNα is upregulated initially (early LASV infection, especially in individuals who will survive infection), IFNα production is suppressed until late time points before death when it is upregulated again [70].

The Z and NP LASV proteins are implicated in specifically inhibiting the expression of IFN-1 (see Meyer et al., 2018 for a recent review [71]). The C-terminal exoribonuclease domain of NP binds to IKKɛ to prevent the activation of IRF3 and IRF7, which are downstream transcription factors that are responsible for inducing the transcription of IFN-1 genes (Figure 1B) [72,73,74]. Specific essential residues have been identified through mutagenesis studies on the LASV NP C-terminal exoribonuclease domain to mediate increased levels of IFN-1 in macrophages and DCs [42,43,75]. The NP residues identified through mutagenesis studies were found to specifically degrade double-stranded RNAs (dsRNAs) that were aberrantly produced and therefore could be recognized by infected cells as pathogen-associated molecular patterns (PAMPs) to induce IFN-1 expression and downstream antiviral responses. The C-terminal exoribonuclease function of NP is unrelated to the role of NP in viral RNA synthesis. The enzymatic function of NP is thought to inhibit the RIG-I-like receptors (e.g., RIG-I and MDA5) which recognize PAMPs and allow control and clearance of viral infection [71,76,77,78,79]. 

Double-stranded RNAs (dsRNAs) can also activate protein kinase R (PKR) upon viral infection to enhance IFN-1 production [80], and this finding has led to the investigation of the role of PKR in IFN-1 production during LASV infection [81,82]. It has been shown that New World (NW) mammarenaviruses [e.g., Junin virus (JUNV)) and Machupo virus (MACV)] can activate PKR, but the Old World (OW) mammarenavirus, LASV, cannot. It has therefore been hypothesized that PKR does not recognize LASV-associated dsRNAs and thus allows LASV to evade innate immune recognition; however, this PKR evasion mechanism is still not entirely understood [82]. The failure of PKR to be activated can partially explain how LASV infection can inhibit the host innate immune system and cellular inflammatory response. The PKR-interacting protein DEAD-box ATP-dependent RNA helicase (DDX3) may play a role to allow LASV to evade PKR activation. Proteomic studies have shown DDX3 protein interactions with mammarenaviral NP, including LASV NP [83]. It has thus been suggested that DDX3-interacting proteins, including MAVS, RIG-I, and PKR, are made unavailable by mammarenaviral NPs and this suppresses IFN-1 expression [83,84].

LASV Z protein can physically interact with elF4E (a eukaryotic translation initiation factor) to inhibit the cellular IFN-1 pathway, which conformationally changes the 5’ cap-binding site of elF4E [33,34,35,36,37,38,85]. The conformational change of elF4E reduces its affinity for cellular 5’ cap structures and reduces the translation of elF4E-dependent cellular proteins (e.g., IRF-7 transcription factor) [37]. During an antiviral immune response, IRF-7 activates IFNα genes via a positive regulatory feedback loop; however, IRF-7 expression is reduced by Z and thus disrupts the activation of IFN-1 expression [86]. In addition to interactions with elF4E, Z protein also antagonizes RIG-I’s dsRNA-induced innate antiviral response. Z protein can disrupt the complex formation between RIG-I and MAVS, which inhibits the production of IFN-1 [36,38]. There is also significant natural sequence diversity in the Z genes of human pathogenic and non-pathogenic mammarenaviruses. It has been recently shown that Z protein sequence variations of two OW human arenaviruses, LASV and Lymphocytic Choriomeningitis Virus (LCMV), serve as more potent inhibitors of the cellular innate immune system as compared to Z protein variants from some other human mammarenaviruses or mammarenaviruses found in naturally infected rodent reservoirs [36,38].

LASV GP has also been shown to play a role in immune evasion during LASV infection. Mammarenaviral GPC is a heavily glycosylated protein, and it is estimated that N-linked glycosylation motifs account for 30% of the total mass of GPC [87]. The 11 N-linked glycans, which are distributed evenly on the surface of LASV GPC with seven glycans on GP1 and four on GP2, play critical roles in the biological function of GP, including cleavage, folding, receptor recognition, and importantly epitope shielding from immune responses [88,89]. Glycan shields have been shown to be an important mechanism of host immune evasion by various viruses, including influenza A, human immunodeficiency virus (HIV), Ebola virus, and LASV. It is hypothesized that these heavily glycosylated viruses (and perhaps other unknown viruses) can evade the host immune system because the glycan chains can shield critical epitopes on the protein surface from being recognized by neutralizing antibodies [89]. Zhu and colleagues found that if the glycosylation motifs of GP were altered by site-directed mutagenesis (N79Q, N99Q, N119Q, N167Q), the proportions of lymphocytes (CD3, CD4/CD3, and CD8/CD3) were not affected, but the proportions of effector lymphocytes (IFN-γ/CD4 and IFN-γ/CD8) increased [89]. They also found increased secretions of IL-2 and IFN-γ (involved in Th1 immune response) after eliminating the glycan chains with N99Q, N119Q, N365Q, N373Q, and N390Q mutations [89]. These results support the idea that N-linked glycans are essential in inhibiting the Th1 immune response and host immune recognition.

## 4. Immune Responses to LF Candidate Vaccines

There are many LF candidate vaccines in preclinical development (Table 1), despite no FDA-approved LF vaccines currently available for human use. The 2017 WHO Target Product Profile (TPP) for LF vaccines emphasizes a high priority for the development of prophylactic vaccines, and optimal candidates should meet WHO-acceptable safety/reactogenicity, and should be single-dose and greater than or equal to 70% efficacy in preventing infection or disease caused by the LASV lineages I-IV, and should be long lasting (greater than or equal to 5 years) [4,90,91]. Most LF vaccine strategies and platforms are based on the LASV GP antigen from the Josiah strain and thus only provide protection against a homologous virus challenge [50,92]. Cellular immunity is more effective in clearing LASV infection as compared to humoral immunity, and so, GP and NP antigens (which induce robust CD4 and CD8 T cell responses) have been chosen for vaccine formulations, especially in viral vector-based and live-attenuated vaccine platforms [59,93]. In the next few paragraphs, we will summarize several known LF vaccine platforms and their in vivo testing results. 

### 4.1. Measles Virus Platform

The INO-4500 vaccine entered the first phase 1 human clinical trial which evaluated the safety, tolerability, and immunogenicity of the LF vaccine candidate in healthy volunteers, and this study was completed in October of 2021; however, results have not been made available yet [94]. The second phase of the clinical trial, which evaluated the dose-ranging in healthy volunteers, started in January 2021 and was expected to be completed in January 2022 [94]. The INO-4500 is a DNA-based vaccine candidate that expresses the LASV GPC [94,95]. The recombinant MV vaccine can be engineered to express antigens from various pathogens and has been shown to be effective, stable, immunogenic, and safe in multiple vaccine studies [96,97] (for reviews see Vrba et al., 2020 [98] and Chen et al., 2021 [99]).

The MV-LASV vaccine expresses the LASV GPC, Z, and/or NP (Josiah strain). The vaccines that expressed either GPC or NP alone provided the best level of protection in cynomolgus macaques against a homologous virus challenge (i.e., challenge with the Josiah strain LASV) [100,101]. MV-LASV was also tested in cynomolgus macaques with different dosages, inoculation routes, and with heterologous challenge [102]. The vaccine was found to induce long-lasting immune responses against diverse LASV strains (lineages II and VII) and elicits T cell responses against GPC and NP [102]. IgG concentrations against LASV GPC and NP were of similar levels, and moderate levels of CD4 and CD8 T cells responding to GPC and NP peptides were found in the blood circulation [102]. LASV IgG was detected in 50% of the immunized macaques after 317 days postimmunization (pi) with a single-dose regiment, and the two-dose regiment significantly improved the humoral response where all macaques had detectable IgG titers one year pi [102]. An in vitro stimulation experiment of PBMCs (using LASV peptides) showed that cytotoxic T cells were still present at one year pi; thus the humoral and cellular responses persisted after one year of immunization with just a single dose of the MV-LASV vaccine [102]. 

All immunized macaques were protected against any tested strains of LASV, even after one year pi when challenged with Josiah strain (homologous challenge) [102]. Challenged macaques had no clinical signs and only transient body temperature increases [102]. One dose was found to have immunity and protection in macaques that was comparable to the two-dose regiment and nAbs were observed in all macaques two weeks after infection and were equally as potent against homo- and heterologous LASV challenge [102]. In addition to studies in macaques, the MV-LASV vaccine was also chosen for a phase 1 human clinical trial. Although the trial was completed in January 2021, the results have yet to be disclosed [101]. It is important to demonstrate whether the MV-LASV V182-001 vaccine can offer protection and, if so, to what extent in those who have received it. If similar results are seen in humans, as what was seen in macaques with a single-dose regiment, MV-LASV vaccine could have a valuable advantage in endemic countries where a two-dose vaccination regiment is more challenging to administer.

### 4.2. Adenovirus Vector-Based Platforms

There have been great progresses made in the development of adenovirus vector-based platforms (e.g., ChAd3, ChAdOx1, ChAd63) as vaccine vectors, which can express antigens of many different pathogens such as influenza and LASV, with some entering human clinical trials (for reviews see Vrba et al., 2020 [98] and Chen et al., 2021 [99]). The vector-based vaccine Ad5 (E1 and E2b-deleted) was able to protect guinea pigs against lethal LASV challenge when it was engineered to express the LASV NP or GPC and was used in a prime-and-boost strategy [103]. In mice, the ChAdOx1 vaccine (expressing LASV GPC Josiah strain) had been shown to be immunogenic and could induce robust LASV-specific CD8 T cell (IFNγ and TNF-α, but low IL-2) and antibody responses [104]. Additionally, guinea pigs were protected againt lethal LASV (guinea pig-adapted Josiah strain) challenge with a single dose of the ChAdOx1 LF vaccine [104]. A prime-and-boost strategy with the ChAdOx1 LF vaccine could significantly increase LASV antigen-specific antibody titers and cleared the virus from challenged animal tissues [104]. Promising results with adenovirus vector-based LASV vaccines call for additional testing in animal models, such as NHPs.

### 4.3. Live Attenuated Virus-Based Platforms

By reassorting the genomic RNA segments of Mopeia virus (MOPV) and LASV (Josiah strain), a live attenuated ML29 vaccine was created [105]. To create a single virion, the L segment of the MOPV and the S segment of the LASV were reassorted (i.e., packaged) into a single particle. This was done by coinfecting LASV and MOPV and then isolating putative reassortments by picking only small plaques. The ML29 was first tested in strain 13 inbred guinea pigs with a single subcutaneous (s.c.) dose 30 days prior to challenge (10^3^ PFU) and was shown to provide a complete level of protection against LASV challenge [106]. Carrion and colleagues also showed that LASV challenge in marmosets caused a fatal, systemic disease [107]. Marmosets are a great model for studying the pathophysiology of LF disease and the effectiveness of vaccines since they share many histological characteristics with humans. It was demonstrated that a single s.c. dosage of ML29 could protect marmosets from LASV challenge (10^3^ PFU), and no animals displayed any clinical symptoms [58]. ML29 immunization of marmosets induced low transient viral replication in tissues with no shedding after low-dose immunization and more extensive virus replication and some (one marmoset) transient shedding in high-dose immunizations [58]. Immunization with ML29 resulted in increased populations of CD14+ and CD3+ T cells, as well as recruitment of CD3+ T cells and over-expression of HLA-DR, P, and Q in the target tissues [58]. These data indicate that there was a significant level of antigen stimulation following ML29 immunization. Additionally, one low s.c. dose of ML29 was enough to induce specific cell-mediated T cell responses (as determined by IFNγ and TNF-α ELISPOT) but induced weak antibody (IgG) responses (determined via IgG ELISA) [58].

When ML29 was used to infect cells in vitro, the L segment-derived and truncated RNA species could be readily detected, additionally when tested in immunodeficient STAT-1-/- mice, immunocompetent mice, and immunocompetent guinea pigs, these truncated RNA species had been shown to play a significant role in influencing the levels of attenuation and immunogenicity, though the underlying mechanism was unknown [108]. The utility of ML29 as a LF vaccine is promising. However, due to infections with HIV-1 and/or other pathogens, a significant portion of the human population in West Africa can be immunosuppressed. According to estimates, LASV seroprevalence is three times greater in HIV-positive individuals, suggesting that LASV and HIV-1 co-infections exist and that both viruses can share the same target human populations [109]. Therefore, before using ML29 in a population-based vaccination strategy, the possibility of negative effects in HIV-positive populations must be thoroughly assessed. It is important to note, however, that when SIV-infected rhesus macaques (which is an excellent model that can recapitulate many aspects of human HIV infection and its viral latency [110]) were vaccinated with the ML29 vaccine, they responded to LASV GP and NP similarly to uninfected macaques [111]. No macaques (SIV-infected and uninfected) developed signs or symptoms of LF disease [111,112], suggesting that ML29 might be safe for use in HIV-infected individuals [4]. However, more research needs to be conducted to ascertain this. 

In addition to ML29, another live-attenuated LASV vaccine candidate has also been developed, called rLASV-GPC/CD. Codon deoptimization (CD) is a technique that reduces gene expression by swapping out the wild-type codons with less-preferred codons across the entire coding sequence of the target gene [113]. The CD strategy has several advantages. For example, reversion to the wild-type sequence is extremely rare because CD depends on the introduction of hundreds of synonymous mutations in the viral gene sequence [114]. Additionally, because CD does not result in amino acid changes that change the viral protein, the CD-containing virus retains the same antigenic epitopes as the wild-type virus [109]. Finally, by combining de novo gene or genome synthesis with reverse genetics, CD viruses can be produced quickly [114]. To date, several successful live attenuated virus vaccines (e.g., poliovirus [113], human respiratory syncytial virus [114], foot-and-mouth disease virus [115], influenza A virus [116], and Zika virus [117]) have been generated using the CD strategy. In particular, the rLASV-GPC/CD vaccine was developed by mutating the GPC gene of LASV, and as such, those codon-deoptimized mutations could interfere with GPC production in the chosen host cells [109].

All strain 13 inbred guinea pigs survived vaccination with rLASV-GPC/CD (one s.c. dose injection) and did not develop any clinical signs of disease, unlike the controls [109]. There was no vRNA detected in the blood or tissues collected from the rLASV-GPC/CD vaccinated animals during any time point of the experiment, however, anti-LASV IgG plasma titers in those animals indicated that they were properly vaccinated with rLASV-GPC/CD. There were no anti-LASV nAbs detected in any of the immunized animals, nor were there any significant histopathological findings or NP antigens detected in the examined organs and tissues, and thus, indicating a complete attenuation of rLASV-GPC/CD in strain 13 guinea pigs. After 30 days pi, the guinea pigs were exposed to a lethal challenge dose of LASV (10^5^ pfu, Josiah strain). Immunized guinea pigs were completely protected from LASV challenge as evidenced by that fact that no vRNA was detected in the blood or tissues at any time point, no histopathological lesions or LASV antigens were detected in the tissues. Additionally, anti-LASV IgG antibody titers were not significantly boosted after LASV exposure, and interestingly, anti-LASV neutralizing antibody titers were not detected which suggested that nAbs did not play a significant role in protection. The same study also showed similar results in the immunized Hartley outbred guinea pigs [109]. Overall, these data show that a single dose of rLASV-GPC/CD was attenuated in and completely protected guinea pigs (strain 13 and Hartley) from LASV challenge and subsequent disease. 

Although ML29, rLASV-GPC/CD, and other live-attenuated vaccines show promising results, an issue with some LASV live-attenuated vaccine candidates is that a relatively high dose is needed to be effective. These high doses of the LASV live-attenuated vaccines could cause significant side effects [109,118] which was observed in a similar platform used to develop the EBOV candidate vaccines [119,120]. Introducing a vaccine that can induce undesired side effects in an area with a poor health care infrastructure could pose a significant obstacle in implementing vaccine campaigns in West Africa. 

### 4.4. Vesicular Stomatitis Virus-Based Platforms

Recombinant VSV (rVSV) vaccine candidates have also shown some promising results. VSV platforms are considered advantageous due to the high level of immunogenicity and the lack of pre-existing immunity in human populations [121]. The rVSV platform was safe as it was genetically modified to lack the glycoprotein (G) gene, which is a significant viral pathogenicity factor [121]. The LASV GP or similar genes from other pathogens, such as EBOV and Marburg (MARV), could be substituted for the VSV G gene [122]. The rVSV Ervbo EBOV vaccine had been evaluated in clinical trials and had been approved for human use by the FDA [122]. Recently, IAVI (a nonprofit scientific research organization) has just started a phase 1 clinical study using the rVSV platform against LASV in Monrovia, Liberia in collaboration with the Coalition for Epidemic Preparedness Innovations (CEPI) [123]. There is no update on the results, but participants would receive one dose of the vaccine and then be followed for a year to determine the safety, tolerability, and the ability for the vaccine to elicit a response against LASV infection [123]. 

Another rVSV vaccine, VSVΔG/LVGPC, has shown promising results in guinea pigs and NHPs. The VSVΔG/LVGPC vaccine expresses GPC of LASV (Josiah strain). When macaques were vaccinated with a single intramuscular (i.m.) dose (2 × 10^7^ pfu) and then challenged 28 days later with LASV (10^4^ pfu), the animals were completely protected against virus challenge [121]. Therer was no evidence of rVSV vector replication or shedding, which further highlights the vaccine’s safety profile [121]. The VSVΔG/LVGPC vaccine induced strong humoral and cellular immune responses in the vaccinated and challenged macaques (*n* = 4) and protected them against a high lethal dose of LASV challenge [121]. Protection in the macaques was associated with CD8 T cell (production of IFNγ and TNF-α) and antibody responses [121]. Although a homologous LASV challenge was used in this investigation, previous research in NHPs and Hartley outbred guinea pigs had demonstrated 100% protection against heterologous LASV challenge [124,125].

The rVSV platform is notable, since it is the first vaccine strategy to show cross-clade protection against all important LASV lineages [126]. Another rVSV vaccine called VesiculoVa is a quadrivalent platform that consists of rVSV vectors expressing filovirus glycoproteins and vectors expressing the glycoprotein of LASV (lineage IV strain). Using a prime-and-boost strategy of the VesiculoVa, cynomolgus macaques were completely protected against three filoviruses (Ebola, Sudan, and Marburg) and a heterologous lineage II LASV challenge [126]. Because rVSV vaccines are replication-competent viruses, further safety-related research must be done before rVSV-based LF vaccinations can be widely used.

### 4.5. Recombinant Vaccinia Virus-Based Platforms

Promising results have been seen with recombinant vaccinia viruses (VACs) that were developed to express LASV NP and GPC genes. In order to study the LASV antigens’ potential as effective vaccine antigens, NHPs were immunized with VAC-LASV candidate vaccines that were developed using the New York Board of Health (NYBH) strain of the vaccinia virus and expressed various LASV NP and GPC protein fragments [56]. In LASV-challenged animals, full-length GPC offered total protection. When challenged with LASV, animals that had received either full-length GPC or full-length S segment vaccinations (i.e., the short segment that contains GPC and NP genes of LASV) displayed dramatically reduced LASV titers [56]. Disease outcome did not correlate with preexisting high-titer antibodies to LASV NP, which is similar to what is seen in patients in Sierra Leone [121]. Instead, cytotoxic T cells have the primary role in protection from LASV challenge, which leads to the conclusion that antibody responses alone do not provide adequate protection [121]. Despite these encouraging outcomes, VAC-based vaccine platforms may still pose a risk to HIV-1-positive (immunosuppressed) patients. As a result, additional research and considerations are needed.

### 4.6. Replicon RNA Viral Vector-Based Platforms

RNA replicon vectors can be generated by in vitro transcription for use in direct RNA immunization [127]. This method of immunizing with replicon RNA vectors has resulted in strong immune responses in various animals and can generate neutralizing antibodies [127]. Kainulainen and colleagues have developed a LASV replicon particle (LASV-VRP) vaccine via reverse genetics technology. Strain 13 inbred guinea pigs were immunized with an s.c. dose (10^7^ FFU) of purified LASV VRPs [128]. No clinical signs were observed in any vaccinated animals prior to challenge. Additionally, in the 28 days leading up to challenge, 4/5 vaccinated guinea pigs developed LASV antibodies [128]. These antibodies were against LASV NP and had no neutralizing activity, as they were not directed against GPC. Twenty-eight (28) days after immunization, guinea pigs were challenged with a s.c. challenge dose (10^4^ FFU) of rLASV (Josiah strain). One dose of LASV-VRP was enough to protect animals from clinical disease and lethal infection [128]. Interestingly, while animals vaccinated 1 day after challenge with a lethal dose of LASV developed clinical disease, all animals survived, and this observed survival benefit is encouraging and suggesting that LASV-VRP can be used as a potential therapeutic vaccine [128]. Intriguingly, as well as developing NP antibodies prior to LASV challenge, NP antibodies were also detected in terminal end-point samples of control animals (no neutralizing activity), thus suggesting that there might be a substantial role for cell-mediated immunity in VRP vaccine-induced protection [128].

The same group of researchers also verified that their LASV-VRP platform could protect strain 13 guinea pigs from homologous virus challenge (LASV Josiah) and could provide complete protection against heterologous virus infection. When animals were vaccinated (s.c.) with LASV-VRP (Josiah strain) or mock infected, and 28 days later were challenged with the Sauderwald strain (clade II) or the N-231 (clade III) [129], the vaccinated animals were completely protected against clinical signs following heterologous challenge, and no viral RNA was found in blood or tissue samples [129]. This study shows a great promise for potential cross-protective vaccine development for LASV and the utility of the VRP platform.

## 5. Immune Competent and Incompetent Animal Models of LF

There are several small animal models for LF disease (e.g., mice and guinea pigs), but only NHPs develop clinical features of LF which mimic those of humans (for in-depth reviews, see Oestereich et al., 2016 [69] and Sattler et al., 2020 [126]) (Table 2). Wild-type immunocompetent mice are naturally resistant to LASV and do not develop LF disease signs, so their use to study disease pathogenesis, therapeutics, and vaccine developments is limited. A breeding colony of wild-caught *M. natalensis* (i.e., the natural host of LASV) has recently been established and developed as a model for studying LASV infection [130]. As expected during experimental infection, *M. natalensis* infected with LASV did not cause persistent level of infection or disease symptoms. However, when *M. natalensis* was infected with Morogoro virus (MORV) (a non-pathogenic arenavirus that shares the same host as LASV) at a young age (i.e., up to two weeks after birth), those animals developed a persistent infection and could transmit the virus horizontally [131]. However, animals older than two weeks at the time of infection could rapidly clear the infection [131]. 

Further research is required since it is possible that the immune system of *M. natalensis* might respond differently to mammarenavirus infection. As further research in this newly established animal colony is needed, it is notable that Tang-Huau and colleagues have recently examined commercially available rat and mouse antibodies against T-cell receptors and effector molecules for potential cross-reaction with *M. natalensis*, and have discovered that the adaptative cellular immune responses of *M. natalensis* lymphocytes to common mitogens (such as phytohaemagglutinin P, lipopolysaccharide, and concanavalin A) are uniquely different from those of laboratory rodents [132]. This immune response variation emphasizes the need to design and improve standards for wild-caught rodent models to appropriately assess the animal’s immunological capabilities as reservoirs for mammarenviral and other relevant infections (for example, *Leishmania* spp., *Yersinia* spp., and *Borrelia* spp.).

While *M. natalensis* could be a valuable model for understanding LASV infection in the future, the current “gold standard” model for studying LASV is rhesus macaques (NHPs). The most extensively researched species for LASV infection is the macaque, which is a good model for pathogenesis studies and for the testing of antivirals and vaccines since it can develop a disease that is similar to clinical symptoms of LF in humans [126]. Macaques experimentally infected with LASV can succumb to viral infection and showed signs of lethargy, rash, fever, anorexia, leukopenia, thrombocytopenia, and high liver enzyme (AST) levels [133,134]. Viremia levels in these NHPs correlate with disease and survival outcomes similar to what is seen in humans [133,134]. Although macaques are the “gold standard” for LASV studies, there are some critical differences between NHP and human LF disease. NHPs show only focal areas of necrosis in the liver, while humans have hepatocellular necrosis [47]. NHPs also have systemic and pulmonary arteritis and increased time to form blood clots (i.e., partial thromboplastin time), both of which are not typical features of human LF disease [135]. Although there are some differences in LF disease presentation, the main hindrance to LASV studies in NHPs is that it requires biosafety level 4 (BSL4) containment, so efficacy studies can be quite costly. The shortage of these animals, their high costs of care, and ethical issues also limit the use of NHPs in LF research.

Small animal models, including mice and guinea pigs, have been developed (for a comprehensive review, see Sattler et al., 2020 [126]) to circumvent those issues with NHPs. Briefly, to name a few, since immune-competent mice are naturally resistant to LASV, mice lacking the receptors for interferon α and β (IFNAR-/- mice) have been created. LASV infection of IFNAR-/- mice can result in a non-lethal acute infection with persistent viremia [126,136,137]. Additionally, chimeric IFNAR-/-B6 (IFNAR-/- mice can be irradiated and then have wild-type C57BL/6′s bone marrow progenitor cells transplanted into them) have also been developed, which can produce a lethal infection model [69,126]. Mice lacking signal transducer and activator of transcription 1 (STAT1-/-) is a promising model for vaccine and therapeutic testing since they are highly susceptible to LASV infection and present a lethal disease with SNHL manifestations [126,138].

Other small animal models of LASV infection include strain 13 inbred and Hartley outbred guinea pigs. LASV-infected inbred strain 13 guinea pigs are a lethal LF model useful for studying pathogenesis and antiviral, vaccine, and immunotherapy candidates [126]. Inbred strain 13 guinea pigs do not require virus adaption (unlike Hartley guinea pigs), but they are not as widely available as Hartleys [126]. Outbred Hartley guinea pigs are susceptible to infection by the Josiah strain LASV and can experience lethal infection in 30%–70% of the infected animals [139]. The Josiah strain must be adapted in Hartley guinea pigs to achieve uniform lethality. However, a LASV clinical isolate (i.e., LF2384) has been shown to be able to achieve uniform lethality in Hartley guinea pigs without adaption [103,140]. This newly developed LF2384-Hartley guinea pig model has shown promises to test adenovirus vector-based LF vaccines [103,140].

In addition to animal models for use with LASV, several surrogate models of LF have been developed with LCMV and Pichinde virus (PICV) to circumvent BSL4 containment needs [141,142]. The WE strain of LCMV can cause lethal disease in rhesus macaques if given intravenously. However, not all studies have shown uniform lethality [143,144]. PICV (a non-pathogenic NW arenavirus) infection (strain CoAn 4763) in strain 13 guinea pigs can cause LF-like disease and therefore can be used as a surrogate BSL2 LF model [126,139]. In Hartley guinea pigs, PICV strain CoAn4763 does not cause uniform lethality unless it is passaged 18 times in guinea pigs to produce the P18 strain [145,146,147], which can show many disease signs and pathologies similar to LASV in humans [141,142]. 

## 6. Future Directions

About 37.7 million Africans are thought to be at risk of LASV infection, based on a recent estimate [6]. This number was determined by examining the species distribution model and the locations of human and animal infections with LASV to generate a probabilistic surface of zoonotic transmission potential across Africa [6]. Despite the high estimate of potential LASV infections, there are currently no effective treatments or vaccinations for this lethal viral disease. Due to the relatively high genetic variation among different LASV lineages and the fact that LF patients show symptoms similar to those of other febrile illnessses, diagnosing LASV can be particularly challenging. There are some LASV diagnostics available (for a review, see Murphy & Ly, 2021 [148]), but clinical settings rarely use laboratory-developed protocols, particularly in areas with limited resources (where LASV is endemic). There is an urgent need to advance current diagnostics for clinical validation and to obtain regulatory permits necessary for clinical application. Future diagnostics must be created to make the technology user-friendly, affordable, and capable of differentiating LASV clinical isolates. Despite the high estimation of potential LASV infections in humans, no effective therapeutics or vaccines are currently available against this deadly virus. Supportive care and ribavirin are the only treatments that have been authorized for use to treat LF (for a review, see Murphy & Ly, 2021 [148]), although the effectiveness of ribavirin varies with the severity of LF and the timing of its clinical application. 

Vaccines with robust T cell responses against LASV glycoproteins have been shown to be protective in animal models. That being said, consideration of inclusion of GPC in its prefusion state in vaccine formulation should be considered, as published studies have shown that mAbs against this property of the viral glycoprotein can be effective in protecting guinea pigs and NHPs against LASV challenge. Few candidate vaccines have advanced through the stages of clinical testing, as the majority are still in preclinical or early clinical stages of development. Vaccine types that are in preclinical development include measles virus-based vaccine (INO-4500), which is in phase 2 clinical trials, and MV-LASV (V182-001), which is in phase 1 clinical trials, adenovirus vector-based (ChAd3, ChAdOx 1, and ChAd63), live-attenuated (ML29 and rLASV-GPC/CD), VSV-based, recombinant vaccinia-based (VAC-LASV), and RNA replicon LF candidate vaccines. A potential issue with many of the vaccines in preclinical development is that many of them require the use of more than one vaccination dose. Repeated vaccination in underdeveloped countries can be a significant challenge, as there is a lack of resources, access to health care facility, and/or personal motivation. For example, according to the WHO, 18.2 million infants did not receive any dose of the Diptheria-Tetanus-Pertussis (DTP) vaccine and an additional 6.8 million were only partially vaccinated with the DTP vaccine [149]; both of these figures point to the potential complications of the prime-boost vaccination strategy. Since there are not many LF vaccines currently in advanced clinical development, more research into the fundamental biology of LASV and the immunological effects it has on the infected individuals will be crucial for understanding viral virulence and pathogenicity. To develop effective therapeutic and/or preventive measures against this lethal human viral disease, more research is needed to identify and describe circulating strains of LASV and the rodent vector populations as well as the immune responses in rodent reservoirs and of humans against the infection.

**Table 1:** Summary of LASV vaccine candidates in preclinical testing.

Vaccine Candidate	Animal Model	Antigen or Virus Strain *	Dose Number	Dose **	Days to Challenge	SurvivalRate (%)	Test Parameter(s)	Date	Reference
Recombinant vesicular stomatitis virus	Guinea pig (strain 13)	GPC and NP	1	1 × 10^6^	28	100	Antibody	2015	[150]
	Cynomolgus macaque	GPC	1	2 × 10^7^–6 × 10^7^	28	100	Antibody and T-cell IFN-gamma	2005, 2015	[121,150]
DNA	Guinea pig (strain 13)	GPC	3	1 × 10^5^	63	100	Antibody and T-cell IFN-gamma	2013	[151]
Recombinant Yellow fever 17D	Guinea pig (strain 13)	GPC-AV	1	1 × 10^5^	21	80	Antibody	2006	[152]
	Guinea pig (strain 13)	GP1 and GP2	2	5 × 10^6^	44	83	Antibody	2011	[153]
Recombinant Mopeia/Lassa fever (ML29)	Guinea pig (strain 13)	GPC and NP	1	1 × 10^3^	30	100	Antibody	2005	[106]
	Marmoset	GPC and NP	1	1 × 10^3^	30	100	Antibody and T-cell IFN-gamma	2008	[58]
rLASV-GPC/CD	Guinea pig (strain 13 and Hartley)	GPC (with codon deoptimization)	2	1 × 10^2^–1 × 10^4^	30	100	Antibody	2020	[109,154]
Vaccinia (NYBH) vectored virus	Rhesus and cynomolgus macaques	GP1, GP2, GPC, and NP	1,2	1 × 10^9^	62-488	90 (GPC/NP vaccines)	Antibody and T-cell IFN-gamma	1989	[155]
Nanocarrier rGP1 encapsulated into polymerase	C57BL/6 mice	GP1–unknown	2	10 µg	14 and 28	0–all animals sacrificed at 28 days, no survival data	Antibody/CD4 T-cell/B cell	2017	[156]
Chimpanzee adenovirus–ChAdOx1-Lassa-GP	Guinea pig (Hartley)	GP	2				Antibody/CD4 T cell		[93]
	Mice and guinea pigs (Hartley)	GPC	1	1 × 10^8^ IU or 3 × 10^8^ IU	28 and 56	100	Antibody/CD4 T cell	2021	[104]
Chimpanzee adenovirus–Ad5 (E1 and E2b deleted)	Mice and guinea pigs (Hartley)	GPC and NP	2	1 × 10^10^ IU	40 and 56	100	Antibody	2019	[103]
Lassa virus-like particles	BALB/c mice	Z, GPC, and NP	2	10 µg	N/A	0–Challenge virus not assessed	Antibody/CD4 T Cell	2010	[17]
Inactivated LASV	Rhesus macaques	Inactivated LASV–Unknown strain	1	1 × 10^4^	108	0	Antibody	1992	[157]
Measles virus (MV) backbone	Cynomolgus macaques	GPC and NP	1	2 × 10^6^ TCID50	37	100	Antibody/CD4 T Cell	2021	[100]
MV-LASV	Cynomolgus macaques	GPC and NP	1 and 2	3000 FFU	37	100	Antibody/CD4 and 8 T cell	2021	[102]
	Humans (Phase 1)	GPC and NP	2 (low and high doses)	Not known		0 -Challenge virus not assessed	Adverse events, injection site reactions, Antibodies, Neutralizing antibodies, and T-cell IFN-gamma	2021	[101]
INO-4500 (DNA-based vaccine)	Humans(Phase 1)	GPC	1-2 intradermal	1 mg followed by EP with CELLECTRA 2000 (electroporation)	N/A	0–Challenge virus not assessed	Adverse events, injection site reactions, Antibodies, Neutralizing antibodies, and T-cell IFN-gamma	2020	[94]
Alphavirus	CBA/J mice	VLPs–GPC genes of clades I and IV, LASV/NIG/LP and LASV/Josiah	21	N/A	Challenge virus not assessed	Challenge virus not assessed	Antibody/CD4 T Cell	2018	[158]
Venezuelan equine encephalitis virus replicon(Alphavirus)	Guinea pig (strain 13)	GPC and NP	3	1 × 10^7^	112	100	Antibody	2001	[159]
Replicon RNA viral vector	Guinea pigs (strain 13/N)	GPC	1 subcutaneous	1 × 10^7^ FFU	28	100 (Homologous and heterologous)	Antibody	2020, 2022	[127,128,129]

* LASV antigens are from the Josiah strain and challenge virus is homologous unless noted otherwise. ** Dose is in PFUs (plaque forming units) unless noted otherwise. IU: infectious units. FFU: Focus forming units.

**Table 2:** Summary of LASV animal models.

Animal Model *	Virus	Disease Signs	Pros	Cons	Reference
*M. natalensis*	LASV	No persistent infection or signs of disease	Evaluation of immunological properties of these animals as reservoirs. Natural host of LASV	Does not develop clinical features of LF. Lack of reagents for species.	[132]
	MORV	Persistent infection and horizontal transmission in young animals. Animals older than two weeks rapidly clear infection.Non-lethal infection model	Evaluation of immunological properties of these animals as reservoirs. Develops persistent infection.	Lack of reagents for species.	[131]
Macaques	LASV	Similar signs of LF disease as humans, viremia levels correlate with disease/survival outcomes.Lethal infection model	“Gold standard” model Develop disease like humans Lethal infection model	Only focal areas of liver necrosis Systemic/pulmonary arteritis and increased partial thromboplastin times Costly and limited supply Ethical concerns	[47,126,134,135,160]
	LCMV	Lethal (intravenous) infection model	BSL2	Non-uniform lethality	[143,144]
IFNAR-/- mice	LASV	Non-lethal acute infection model with persistent viremia	Susceptible to disease unlike other mouse models	Non-lethal disease model	[126,136,137]
chimeric IFNAR-/-B6	LASV	Lethal infection model	Susceptible to disease unlike other mouse models Lethal disease model SNHL manifestations		[69,126]
Strain13 Guinea pigs	LASV	Lethal infection model	Do not require virus adaption	Not widely available	[126]
	PICV	Lethal infection model	BLS2 Smaller animal model than LCMV with macaques	Requires adaption (at least four viral passages)	[126,161]
Hartley Guinea pigs	LASV	Lethal infection model (30–70% of animals with Josiah strain)	More readily available	Requires adaption	[103,126,140,161]
	LASV (LF2384)	Lethal infection model (30–70% of animals)	More readily available No adaption required	Requires LF2384 isolate of LASV	[103,140]
	PICV	Lethal infection model	BLS2 Smaller animal model than LCMV with macaques More readily available than strain 13 guinea pigs	Requires adaption (at least 18 viral passages)	[141,142,145,146,147]

* Scientific name of species is italicized.

## Data Availability

No new data were created or analyzed in this study. Data sharing is not applicable to this article.
