# Correction: Murphy, H.; Ly, H. Understanding Immune Responses to Lassa Virus Infection and to Its Candidate Vaccines. *Vaccines* 2022, *10*, 1668

**DOI:** 10.3390/vaccines12080909

**Published:** 2024-08-12

**Authors:** Hannah Murphy, Hinh Ly

**Affiliations:** Comparative & Molecular Biosciences Graduate Program, Department of Veterinary & Biomedical Sciences, College of Veterinary Medicine, University of Minnesota, Twin Cities, St. Paul, MN 55108, USA; murp1625@umn.edu

The authors would like to make the following corrections to this published paper [1].

In the original publication [1], there was a mistake in the first column of Table 1, published as “INO-4500 (MV backbone)”. The corrected notation for this should be “INO-4500 (DNA-based vaccine)”.

There was an error in a sentence in the first paragraph of Section *4.1. Measles Virus Platform*, published as “The INO-4500 is a DNA-based vaccine candidate that expresses the LASV GPC and is based on the Schwarz strain of the measles virus (MV) [94,95]”, which should be changed to the following version: “The INO-4500 is a DNA-based vaccine candidate that expresses the LASV GPC [94,95]”. The first sentence in the second paragraph of Section 4.1, “In addition to INO-4500, another vaccine candidate has been developed using the Schwarz MV vaccine platform, MV-LASV (V182-001, MeV-NP)”, should be removed.

The authors state that the scientific conclusions are unaffected. This correction was approved by the Academic Editor. The original publication has also been updated.
